# Quorum Quenching in a Novel *Acinetobacter* sp. XN-10 Bacterial Strain against *Pectobacterium carotovorum* subsp. *carotovorum*

**DOI:** 10.3390/microorganisms8081100

**Published:** 2020-07-23

**Authors:** Wenping Zhang, Qingqing Luo, Yiyin Zhang, Xinghui Fan, Tian Ye, Sandhya Mishra, Pankaj Bhatt, Lianhui Zhang, Shaohua Chen

**Affiliations:** 1 State Key Laboratory for Conservation and Utilization of Subtropical Agro-bioresources, Guangdong Province Key Laboratory of Microbial Signals and Disease Control, Integrative Microbiology Research Centre, South China Agricultural University, Guangzhou 510642, China; 20191047008@stu.scau.edu.cn (W.Z.); qingqingluo777@163.com (Q.L.); cheungyikwan@163.com (Y.Z.); fxhscau@163.com (X.F.); 20182047012@stu.scau.edu.cn (T.Y.); sandhyamanshi@gmail.com (S.M.); pankajbhatt.bhatt472@gmail.com (P.B.); lhzhang01@scau.edu.cn (L.Z.); 2 Guangdong Laboratory for Lingnan Modern Agriculture, Guangzhou 510642, China

**Keywords:** quorum sensing, quorum quenching, *Acinetobacter* sp. XN-10, *N*-acyl homoserine lactones, *Pectobacterium carotovorum* subsp. *carotovorum*, biocontrol

## Abstract

Quorum sensing (QS) is a cell density-dependent mechanism that regulates the expression of specific genes in microbial cells. Quorum quenching (QQ) is a promising strategy for attenuating pathogenicity by interfering with the QS system of pathogens. *N*-Acyl-homoserine lactones (AHLs) act as signaling molecules in many Gram-negative bacterial pathogens and have received wide attention. In this study, a novel, efficient AHL-degrading bacterium, *Acinetobacter* sp. strain XN-10, was isolated from agricultural contaminated soil and evaluated for its degradation efficiency and potential use against QS-mediated pathogens. Strain XN-10 could effectively degrade *N*-(3-oxohexanoyl)-L-homoserine lactone (OHHL), *N*-hexanoyl-L-homoserine lactone (C6HSL), *N*-(3-oxododecanoyl)-L-homoserine lactone (3OC12HSL), and *N*-(3-oxooctanoyl)-L-homoserine lactone (3OC8HSL), which all belong to the AHL family. Analysis of AHL metabolic products by gas chromatography–mass spectrometry (GC-MS) led to the identification of *N*-cyclohexyl-propanamide, and pentanoic acid, 4-methyl, methyl ester as the main intermediate metabolites, revealing that AHL could be degraded by hydrolysis and dehydroxylation. All intermediates were transitory and faded away without any non-cleavable metabolites at the end of the experiment. Furthermore, strain XN-10 significantly attenuated the pathogenicity of *Pectobacterium carotovorum* subsp. *carotovorum* (*Pcc*) to suppress tissue maceration in carrots, potatoes, and Chinese cabbage. Taken together, our results shed light on the QQ mechanism of a novel AHL-degrading bacterial isolate, and they provide useful information which show potential for biocontrol of infectious diseases caused by AHL-dependent bacterial pathogens.

## 1. Introduction

*Pectobacterium carotovorum* subsp. *carotovorum* (*Pcc*), formerly known as *Erwinia carotovora* subsp. *carotovora*, is a pathogen with a broad host range. It causes soft rot disease in a variety of cruciferous crops during cultivation, post-harvest processing, and storage [1]. Soft rot is an epidemic, worldwide, and severe disease in many valuable commercial vegetables, such as potatoes (*Solanum tuberosum*), Chinese cabbage (*Brassica pekinensis*), carrots (*Daucus carota*), etc., causing significant economic losses [2,3,4]. To reduce the effects of soft rot, some plant extracts have been developed to inhibit the pathogenic strain *Pcc* [5]. At the same time, many chemicals have also been used to control or kill pathogenic bacteria, such as copper sulphate, sodium hypochlorite, and antibiotics [6]. However, because of the adverse impacts of chemicals on the environment and human health, they are no longer recommended for use [7,8]. Therefore, quorum quenching (QQ) based on the disruption of quorum sensing (QS) has been put forward and regarded as a promising strategy to combat pathogenic infection [9].

QS is a widespread phenomenon in microorganisms, and some QS systems have been elucidated in detail [10]. Bacterial QS involves self-produced extracellular QS signal molecules, which can accumulate in a local environment to levels that are required to activate the transcription of specific virulence genes and other biological activities [11]. A range of chemical signal molecules are essential components of the communication system, such as *N*-acyl-homoserine lactones (AHLs) [12], 3-hydroxypalmitic acid methyl ester (3-OH-PAME) [13], 2-heptyl-3-hydroxy-4(1*H*)-quinolone (PQS) [14], autoinducer-2 (AI-2) [15], diffusible signal factor (DSF) [16], and 2-(2-hydroxyphenyl)-thiazole-4-carbaldehyde (IQS) [17]. Novel QS molecules need to be discovered to understand their impact in controlling the pathogens [18].

Among them, the density-dependent control of virulence factors modulated by freely diffusible AHL inter-cellular signaling molecules has been the most extensively studied area and has become a well-established trait of some plant and animal pathogens [10]. Different bacteria produce different AHLs, but they all contain the same homo-serine lactone ring. They differ in acyl chain length (4–18 carbon atoms) or in the substituent group at the carbon 3 position [18,19,20].

AHL-mediated QS regulates the expression of virulence factors of the plant pathogen *Pcc* [21]. Extensive tissue maceration is a specific symptom of soft rot disease caused by *Pcc*, which is attributed to the certain cell wall-degrading exoenzymes closely related to pathogenicity [22]. These exoenzymes, including pectate lyase (Pel), pectin lyase (Pnl), polygalacturonase (Peh), cellulase (Cel), and protease (Prt) are modulated by an AHL QS system, through the signal molecule *N*-(3-oxohexanoyl)-L-homoserine lactone (OHHL) [22]. OHHL is a member of the AHL family and is synthesized by the product of the expl/carl gene and used by *Pcc* [23].

The bacterial QS system can be disrupted by a wide variety of pathways, collectively known as QQ [24]. This promising strategy blocks bacterial cell–cell communication by inhibiting the synthesis of signal molecules, by enzyme-catalyzed degradation, or via modification of extracellular signals, and it impedes the binding of signaling molecules to receptor proteins in QS pathways, through traditional killing methods [25,26,27]. There are two types of QS inhibitors (QSIs), including small-molecule QSIs, extracted from natural resources or obtained by chemical synthesis, and quorum quenching enzymes, which is the most feasible and applicative QQ pathway [28]. To discover QQ enzymes from vast bacterial sources, many strains have been unveiled in various species of *Firmicutes*, *Actinobacteria*, and *Proteobacteria* [29]. Meanwhile, these previous studies indicated that these QQ strains could serve as biocontrol agents to protect hosts and combat bacterial diseases in aquaculture and agriculture.

Therefore, the objectives of the present study were to isolate and characterize a novel, high-efficiency QQ strain, to investigate its AHL degradation capacity and degradation mechanisms, and to evaluate its potential for the biocontrol of soft rot diseases caused by *Pcc*. This study enriches the microbial pool of QQ candidates. It provides practical, useful, and harmless biocontrol agents to protect plants from several infectious diseases caused by AHL-dependent pathogens.

## 2. Materials and Methods

### 2.1. Chemicals and Plants

*N*-(3-Oxohexanoyl)-L-homoserine lactone (3OC6HSL, OHHL), *N*-hexanoyl-L-homoserine lactone (C6HSL), *N*-(3-oxododecanoyl)-L-homoserine lactone (3OC12HSL), and *N*-(3-oxooctanoyl)-L-homoserine lactone (3OC8HSL) were purchased from Sigma Aldrich Chemicals Pvt. Ltd. (Shanghai, China) and dissolved in acetonitrile to make a stock concentration of 100 mmol·L^−1^. The antibiotics ampicillin (Amp), carbenicillin (Carb), streptomycin (Str), tetracycline (TC), gentamicin (Gen), chloramphenicol (Cm), kanamycin (Kan), and neomycin (Neo) were used in antimicrobial susceptibility tests, and X-gal was purchased from Dingguo Biology (Guangzhou, China). Healthy potato (*Solanum tuberosum*), Chinese cabbage (*Brassica pekinensis*), and carrot (*Daucus carota*) plants were purchased from a local market (Guangzhou, China) and selected for the experiments.

### 2.2. Bacterial Strains and Media

*Pectobacterium carotovorum* subsp. *carotovorum (Pcc)* strain Z3-3, *Escherichia coli* DH5α, and *Bacillus thuringiensis* subsp. *israelensis* B23 served as plant pathogen, negative control, and positive control, respectively, in in vitro co-culture assays. A biosensor strain, *Agrobacterium tumefaciens* NT1, was used to detect AHLs contained in samples. All strains were provided by the Integrative Microbiology Research Centre, South China Agricultural University, China, and were routinely grown in Luria-Bertani medium (LB) [composition (g·L^−1^): yeast extract 5.0, tryptone 10.0, NaCl 10.0]. AHL detection was performed on mineral medium (MM) [composition (g·L^−1^): (NH_4_)_2_SO_4_ 2.0, MgSO_4_·7H_2_O 0.2, CaCl_2_ 0.01, FeSO_4_ 0.005, MnCl_2_ 0.002, K_2_HPO_4_ 10.5, KH_2_PO_4_ 4.5, pH 6.5]. The degradation efficiency was analyzed in mineral salt medium (MSM) [composition (g·L^−1^): (NH4)_2_SO_4_ 2.0, Na_2_HPO_4_·12H_2_O 1.5, KH_2_PO_4_ 1.5, MgSO_4_·7H_2_O 0.2, CaCl_2_·2H_2_O 0.01, FeSO_4_·7H_2_O 0.001, pH 6.5]. Solid medium was prepared from a liquid medium containing 2.0% (*w/v*) agar. Both media were autoclaved at 121 °C for 20 min.

### 2.3. Isolation and Screening of AHL-Degrading Bacteria

To isolate AHL-degrading bacteria, approximately 300 g of soil samples from Guangzhou City, Guangdong Province, China were collected for further study. The isolation procedure was carried out as follows: Each soil sample (5 g) was added to MSM medium containing 5 μmol·L^−1^ OHHL for enrichment culture at 30 °C and 200 rpm for 7 days. After 7 days, 10% of the inoculum was transferred to fresh MSM containing OHHL (10 μmol·L^−1^) and cultivated under the same conditions for another 7 days. Following the same method, the concentration of OHHL was continuously increased to 80 μmol·L^−1^. The final suspension was serially diluted (10^−1^–10^−8^) and spread on LB agar plates. After incubation at 30 °C for 24–72 h, colonies with different forms were selected and transferred to fresh LB agar plates for purification [30]. The procedure was repeated until pure strains were obtained. These strains were stored at −80 °C after isolation for further experiments.

Cell isolates were harvested by centrifugation and inoculated in MSM containing OHHL as the sole source of carbon at 30 °C and 200 rpm. After 24 h cultivation, 5 μL of the culture was inoculated on top of the MM agar strip (1 cm width) containing X-gal (40 μg·mL^−1^), and then the AHL biosensor strain, *A. tumefaciens* NT1, was spotted at progressively further distances from these mixtures by placing the MM agar strip in a dark incubator maintained at 28 °C and observing phenomenon after 24 h [27,31,32]. To screen for bacteria possessing higher degradation activity, different concentrations (5, 10, 15, 20, 25, and 30 μmol·L^−1^) of OHHL in MSM medium were set. The most potent bacterium was selected and named as XN-10 for further study.

### 2.4. Identification and Antimicrobial Susceptibility of QQ Strains

The morphology characteristics of strain XN-10 were observed on LB plates after 24–48 h cultivation and slides with a scanning electron microscope (EVO MA 15, Zeiss, Oberkochen, Germany). The whole genome of strain XN-10 was obtained by using an Easy Pure Bacteria Genomic DNA Kit (Shanghai Invitrogen Technology Co. Ltd., Shanghai, China). The genus of bacterial isolates was determined by sequencing analysis of the 16S ribosomal RNA genes amplified using PCR primer pairs 27-F: 5′-AGAGTTTGATCCTGGCTCAG-3′ and 1492-R: 5′-GGTTACCTTGTTACGACTT-3′ as described previously [33]. After purification, the PCR-amplified product was sequenced with Shanghai Invitrogen Technology Co. Ltd., China. A similarity analysis of 16S rDNA was conducted with the Basic Local Alignment Search Tool (BLAST) from the National Center for Biotechnology Information (NCBI) database (https://blast.ncbi.nlm.nih.gov/Blast.cgi). Multiple nucleotide sequence alignment and phylogenetic analyses were performed in MEGA 5.0.

The antimicrobial susceptibility of strain XN-10 was tested to further investigate its biocontrol potential and biological properties. Strain XN-10 was activated overnight and inoculated into LB medium containing antibiotics of different concentrations, which was then cultured at 30 °C and 200 rpm for 24 h. Antibiotics (Amp, Gen, Kan, NeO, Carb, Str, Cm, and Tc) were used in this experiment at concentrations of 5, 10, 20, 50, 100, 150, 200, 250, 300, 350, and 400 μg·mL^−1^. The growth of XN-10 in the different antibiotic concentrations was detected with an ultraviolet spectrophotometer at 600 nm [34,35]. Three replicates were prepared for each treatment.

### 2.5. AHL Degradation Assay

To investigate the relationship between OHHL degradation and growth of strain XN-10, cells of strain XN-10, obtained from the overnight cultures after centrifuging at 10,000 rpm for 1–2 min, were inoculated onto 20 mL MSM medium containing OHHL at a final concentration of 0.5 mmol·L^−1^. Samples were collected at the same interval, and strain growth was measured at OD_600_ after cultivation at 30 °C and 200 rpm. The remaining OHHL was extracted from the supernatant with ethyl acetate and detected by high-performance liquid chromatography (HPLC). Three replications were performed for each treatment, and samples without strain XN-10 served as control. In addition, the abilities of strain XN-10 to degrade other AHLs (C6HSL, 3OC12HSL, and 3OC8HSL) were also determined. Samples of different treatments were collected, and degradation was performed as described above.

Different AHLs were quantified using a Waters e2695 HPLC system equipped with a Phenomenex C_18_ reversed-phase column (250 μm × 4.6 mm × 5 μm) and array detection at 210 nm. The mobile phase, a mixture of acetonitrile and water (30:70, *v*/*v*), was employed at a flow rate of 0.5 mL·min^−1^ [27].

### 2.6. In Vitro Assay of Strain XN-10 against Pcc

Biocontrol assays were performed on potato (*Solanum tuberosum*), Chinese cabbage (*Brassica pekinensis*), and carrot (*Daucus carota*) plants to assess the potential of strain XN-10 against *Pcc* strain Z3-3. Potato tubers, carrot tubers, and Chinese cabbage were firstly washed with tap water, then surface sterilized by sequential immersion in 44% sodium hypochlorite and 75% ethanol for 60 s. Finally, potato and carrot tubers were cut into slices with a thickness of about 0.3 cm, and Chinese cabbage stems were cut into small rectangular pieces of about 6 cm × 4 cm. These slices and stems were air-dried before inoculation.

*B. thuringiensis* subsp. *israelensis* B23 is a well-known biocontrol bacterium possessing AHL-degrading activity. *E. coli* DH5α has no AHL-degrading activity. Four treatments were designed in this experiment: (1) untreated plant slices served as blank control; (2) plant slices treated with Z3-3 and *E. coli* DH5α served as negative control; (3) plant slices treated with Z3-3 and B23 served as a positive control; and (4) plant slices treated with Z3-3 and XN-10 served as the experimental group. All overnight cultures of strains in this experiment were centrifuged to obtain cells. Cells were resuspended in sterile phosphate-buffered saline (PBS). To perform the assay, colony-forming units (CFUs) of XN-10, *E. coli* DH5α, and B23 strains were adjusted to 1.86 × 10^11^ CFU·mL^−1^, and the CFU of pathogen Z3-3 was adjusted to 1.2 × 10^5^ CFU·mL^−1^. Strain Z3-3 was separately mixed with strain XN-10, *E. coli* DH5α, and B23 at the same volume, and control groups were only treated with pure pathogen Z3-3. Then, 2 μL of each mixture was injected into biocontrol materials (potato slices, carrot slices, and Chinese cabbage). All treatments consisted of at least three slices, and experiments were repeated at least three times. Disease severity was evaluated numerically after 24 h of incubation at 28 °C. To quantify the severity of the disease, the proportion of the macerated region was calculated in comparison to pre-inoculated tissue [27].

### 2.7. Identification of AHL Metabolic Products

To identify the metabolic products from AHL degradation and the metabolic pathway, the XN-10 strain was grown in MSM medium supplemented with 1 mmol·L^−1^ AHL. Non-inoculated samples containing the same amount of AHL served as control. AHLs of all samples collected at regular intervals were extracted three times with ethyl acetate, followed by drying and resuspension in methanol for gas chromatography–mass spectrometry (GC-MS) analysis [28]. The samples were detected by GC-MS (Model 7890B/5977B, Agilent Technologies Inc., Santa Clara, CA, USA) equipped with an Agilent HP-5MS capillary column (30 m × 250 μm × 0.25 μm) in scan mode and split mode (10:1). Temperatures of the inlet, ion source, GC-MS interface, and quadrupole were maintained at 250 °C, 230 °C, 280 °C, and 150 °C, respectively. The following temperature programming was applied during detection: 150 °C isothermal for 2 min; 25  °C/min up to 280  °C, then isothermal for 3 min. To identify degradation products, mass spectrograms of chemicals contained in samples were matched with that of standard compounds from the National Institute of Standards and Technology (NIST, Gaithersburg, MD, USA) library database.

### 2.8. Statistical Analysis

Each treatment was repeated three times to verify the data statistically. Experimental data were analyzed by one-way analysis of variance (ANOVA), and means were compared according to Bonferroni’s multiple comparison test in Graphpad Prism (version 6). Statistical significance was determined by Tukey’s HSD Test at *p* < 0.05 to examine specific differences between groups.

## 3. Results

### 3.1. Screening, Identification, and Antimicrobial Susceptibility of AHL-Degrading Strains

**Figure 1:**
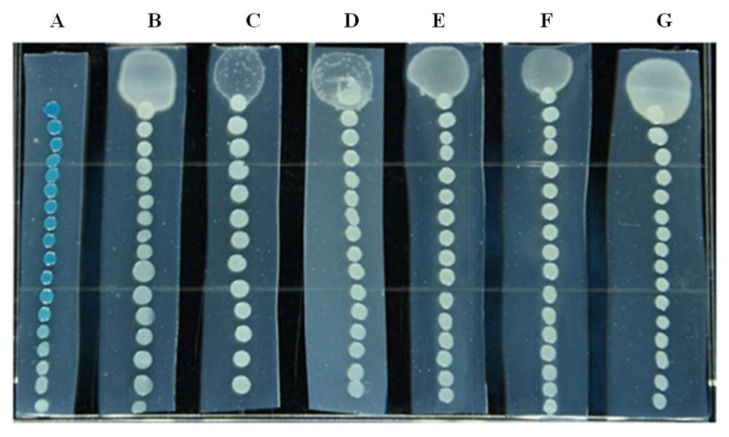
Degradation activity of *N*-acyl-homoserine lactone (AHL) by strain XN-10. The degradation activity of AHL by strain XN-10 was tested using various concentrations of *N*-(3-oxohexanoyl)-L-homoserine lactone (OHHL) (i.e., 5, 10, 15, 20, 25, and 30 μmol·L^−1^) (**B**–**G**) compared to a blank control (**A**) only containing 30 μmol·L^−1^ of OHHL. The residual content of OHHL in the sample was determined according to the distance of the report strain on the agar strip from the top to blue. The columns containing the various concentrations of OHHL did not turn blue, indicating that the OHHL was completely degraded by strain XN-10.

In this study, several AHL-degrading strains were isolated from soil samples, and the one possessing the highest degradation efficacy was designated as XN-10. As shown in Figure 1, all the AHL, contained in the MSM medium at the concentration of 5–30 μmol·L^−1^, was utilized by strain XN-10 within 24 h.

**Figure 2:**
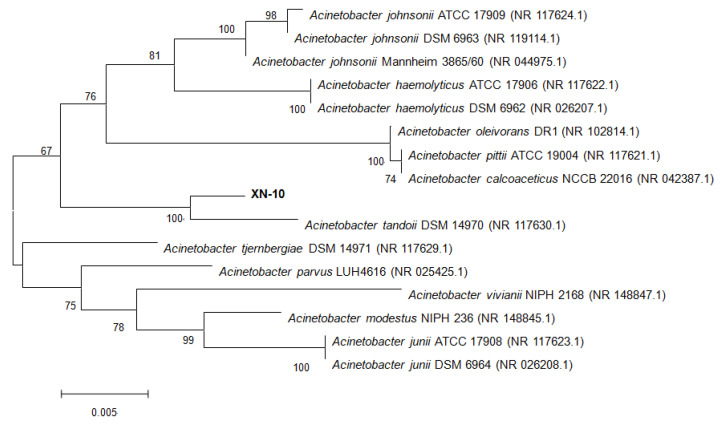
Phylogenetic analysis based on the 16S rRNA sequence of strain XN-10 and other representative *Acinetobacter* strains. The phylogenetic tree was constructed with the neighbor-joining (NJ) method. Numbers in parentheses represent the sequence accession numbers in GenBank. Numbers at the nodes indicate bootstrap values from the neighborhood-joining analysis of 1000 resampled datasets. Bars represent sequence divergence.

The colony of isolate XN-10 was white-yellow, round, small, convex, and smooth with neat edges, as determined on LB solid medium after 24 h cultivation (Figure S1a). Scanning electron microscopy characterized strain XN-10 as a short, rod-shaped (0.4–0.8 × 1.1–1.6 μm), and non-flagellated bacterium (Figure S1b). BLAST results of the 16S rDNA sequence analysis revealed that strain XN-10 showed a high similarity (≥99%) to different *Acinetobacter* strains. Strain XN-10 was closely clustered with the *Acinetobacter* group in a phylogenetic tree (Figure 2). In combination with the morphology and 16S rDNA gene analysis, strain XN-10 was tentatively identified as *Acinetobacter* sp.

**Figure 3:**
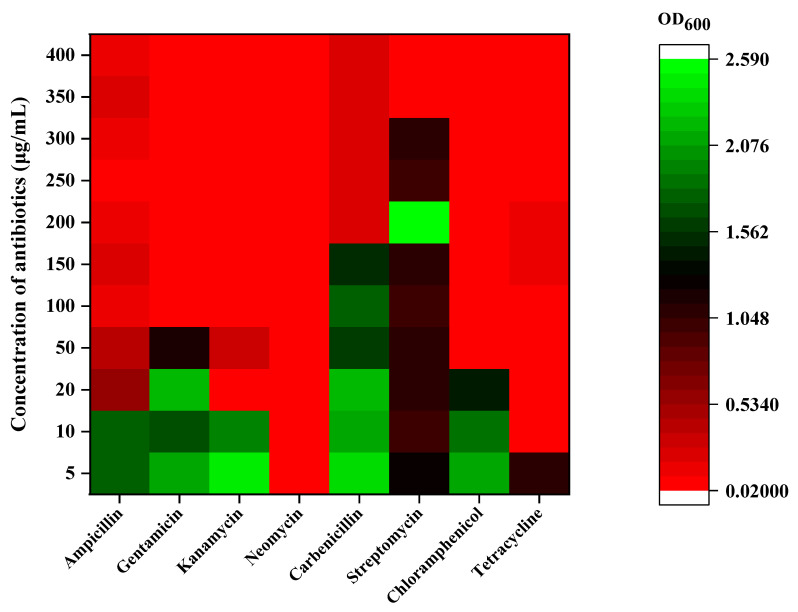
Antibiotic sensitivity of strain XN-10. This figure shows the results of the sensitivity of strain XN-10 to various concentrations of each antibiotic. The green in the figure indicates that the strain has strong resistance to this concentration of antibiotics, and the red indicates that the strain is susceptible to this concentration of antibiotics.

Antimicrobial susceptibility of strain XN-10 was determined in a further study. The sensitivity levels of strain XN-10 against different antibiotics are indicated in Figure 3. The resistance of strain XN-10 to streptomycin (Str) was noted up to a concentration of 300 μg·mL^−1^. Strain XN-10 exhibited resistance against carbenicillin (Carb) up to a concentration of 250 μg·mL^−1^, 50 μg·mL^−1^ against chloramphenicol (Cm) and gentamicin (Gen), 10 μg·mL^−1^ against kanamycin (Kan), and 200 μg·mL^−1^ against ampicillin (Amp). Resistance of strain XN-10 against neomycin sulphate (NeO) and tetracycline (Tc) reached up to 50 μg·mL^−1^. Three replicates were conducted for each treatment. The growth of strain XN-10 against antibiotics at different concentrations was measured by absorbance at 600 nm.

### 3.2. AHL Degradation Abilities of Strain XN-10

**Figure 4:**
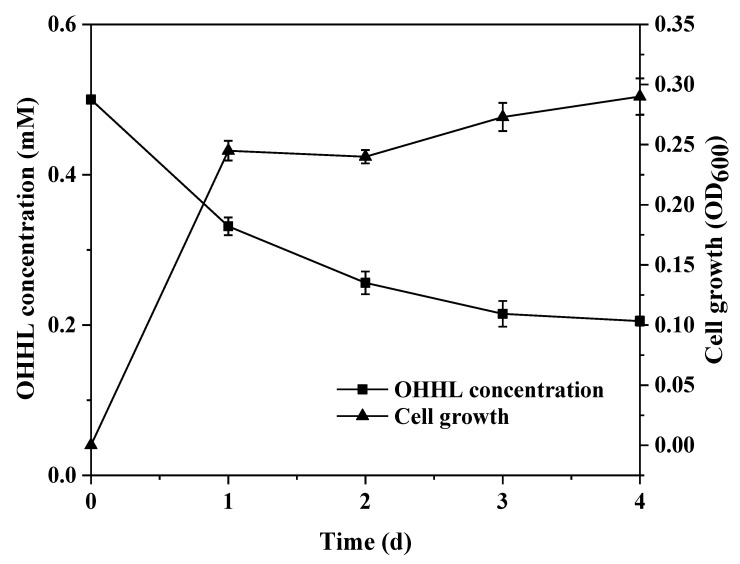
The relationship between *N*-(3-oxohexanoyl)-L-homoserine lactone (OHHL) degradation and growth of strain XN-10. Symbols: OHHL concentration; and cell growth. Values represent the mean of three repeats. Each experiment was conducted with three replicates. Bars indicate standard deviation of the mean.

**Figure 5:**
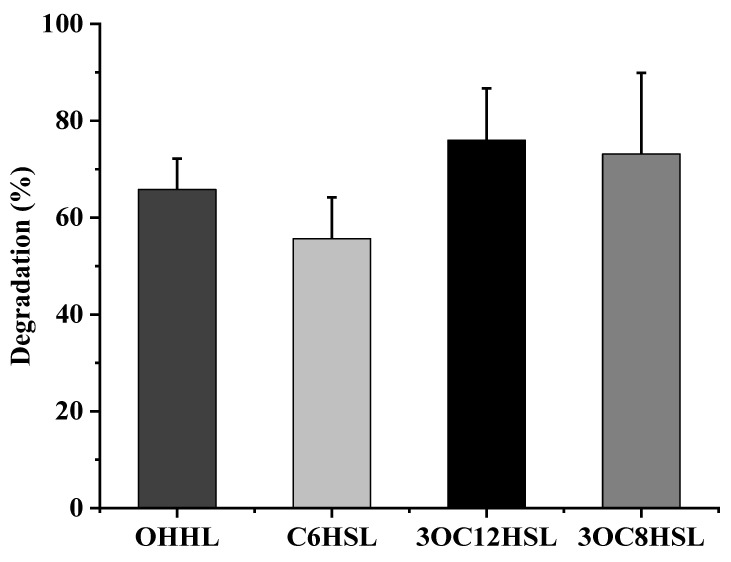
The degradation abilities of strain XN-10 to different AHL signal molecules. OHHL: *N*-(3-oxohexanoyl)-L-homoserine lactone; C6HSL: *N*-hexanoyl-L-homoserine lactone; 3OC12HSL:*N*-(3-oxododecanoyl)-L-homoserine lactone; 3OC8HSL: *N*-(3-oxooctanoyl)-L-homoserine lactone.

To disclose the relationship between AHL degradation and growth of isolate XN-10, an AHL degradation assay was performed in vitro, and the remaining AHL was measured with HPLC using the method described above. Figure 4 presents the relationship between OHHL degradation and the growth of XN-10. Cell growth spiked during the initial growing period (0–1 days), and the residual amount of OHHL reduced significantly. Finally, 65.8% OHHL was utilized by strain XN-10 on the 4th day. Figure S2 revealed that residual OHHL gradually decreased with time, and OHHL degradation reached up to 11.0%, 15.0%, 19.6%, and 65.8% after 1, 2, 3, and 4 days, respectively. In addition, strain XN-10 was capable of degrading various AHLs including OHHL, C6HSL, 3OC12HSL, and 3OC8HSL. As shown in Figure 5, the degradation of strain XN-10 to different AHLs is above 57.0%, and the degradation effect of 3OC12HSL is the best, and the total degradation can reach 76.0% within 4 days.

### 3.3. Potential Use of Strain XN-10 against Pcc Infections

**Figure 6:**
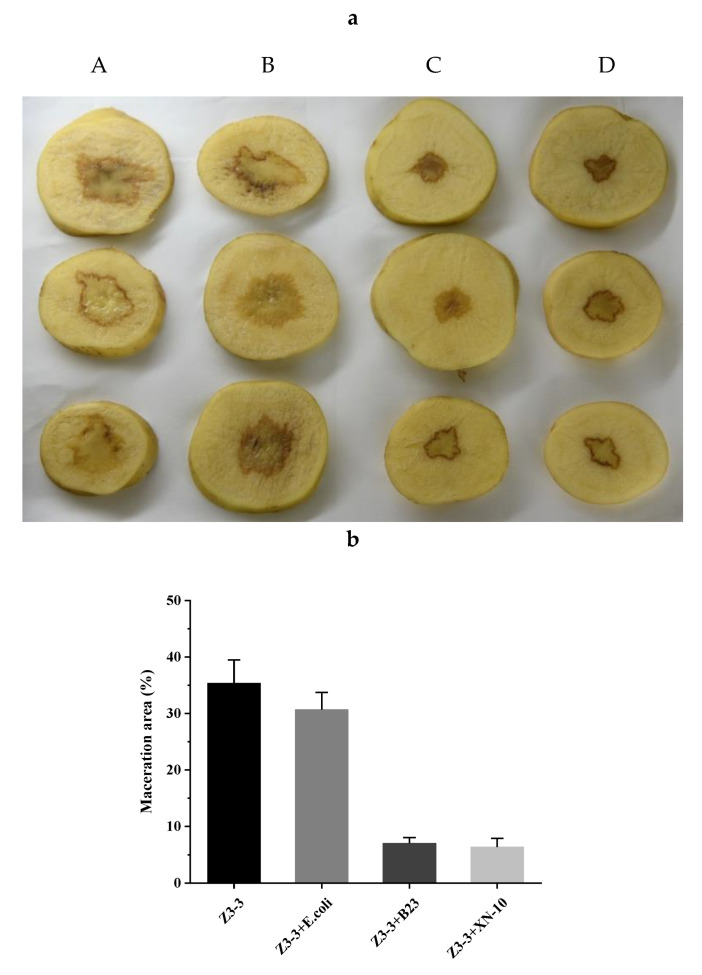
Biocontrol test of strain XN-10 against *Pectobacterium carotovorum* subsp. *carotovorum* (*Pcc*) on potato slices under laboratory conditions. (**a**) Attenuated maceration of *Pcc* strain Z3-3 by strain XN-10 on potatoes. A: inoculation of Z3-3 alone; B: co-inoculation of Z3-3 and *Escherichia coli* DH5α; C: co-inoculation of Z3-3 and *Bacillus thuringiensis* subsp. *israelensis* B23; and D: co-inoculation of Z3-3 and strain XN-10. (**b**) Tissue maceration rates in each treatment.

**Figure 7:**
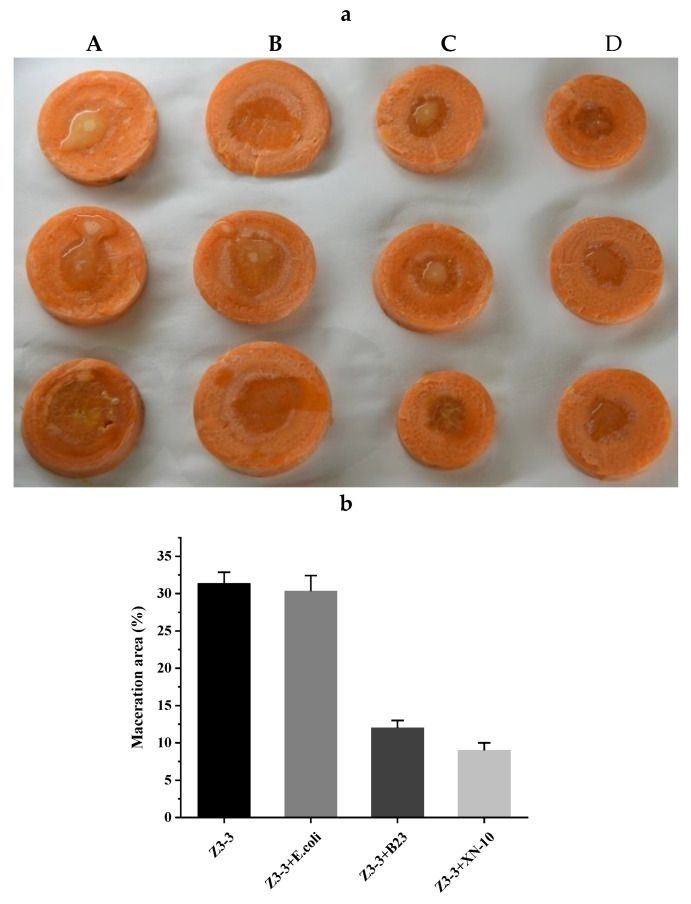
Biocontrol test of strain XN-10 against *Pectobacterium carotovorum* subsp. *carotovorum* (*Pcc*) on carrot slices under laboratory conditions. (**a**) Attenuated maceration of *Pcc* strain Z3-3 by strain XN-10 on carrots. A: inoculation of Z3-3 alone; B: co-inoculation of Z3-3 and *Escherichia coli* DH5α; C: co-inoculation of Z3-3 and *Bacillus thuringiensis* subsp. *israelensis* B23; and D: co-inoculation of Z3-3 and strain XN-10. (**b**) Tissue maceration rates in each treatment.

**Figure 8:**
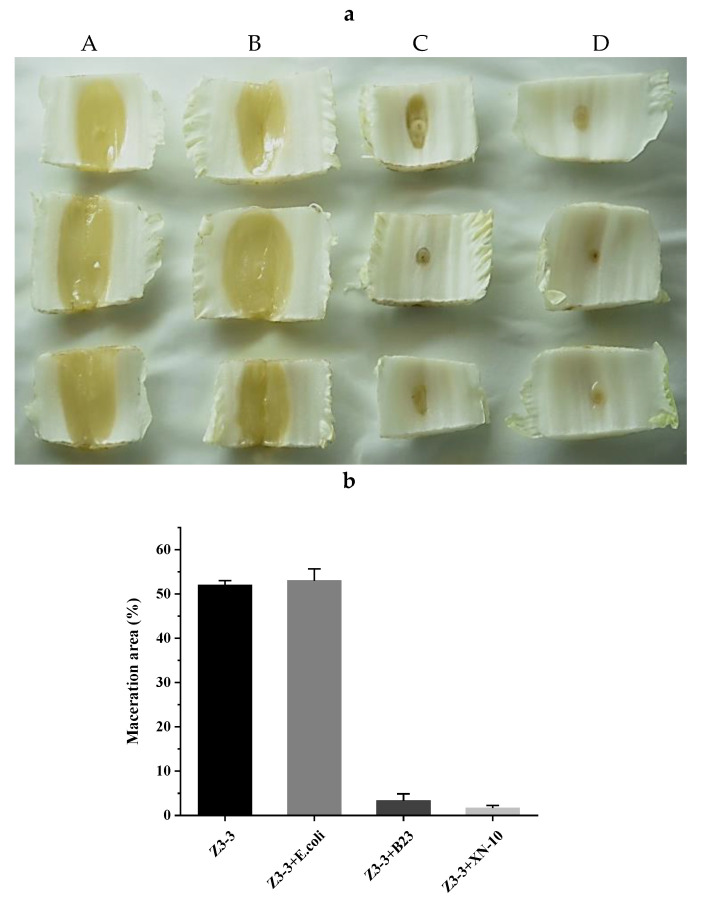
Biocontrol test of strain XN-10 against *Pectobacterium carotovorum* subsp. *carotovorum* (*Pcc*) on Chinese cabbage under laboratory conditions. (**a**) Attenuated maceration of *Pcc* strain Z3-3 by strain XN-10 on Chinese cabbage. A: inoculation of Z3-3 alone; B: co-inoculation of Z3-3 and *Escherichia coli* DH5α; C: co-inoculation of Z3-3 and *Bacillus thuringiensis* subsp. *israelensis* B23; and D: co-inoculation of Z3-3 and strain XN-10. (**b**) Tissue maceration rates in each treatment.

A biocontrol assay was performed on potato (Figure 6), carrot (Figure 7), and Chinese cabbage (Figure 8) plants to evaluate the potential use of XN-10 against *Pcc* Z3-3. The results indicated that strain XN-10 possessed competence against *Pcc* Z3-3. Every group had four treatments: controls treated only with Z3-3 (A), negative control treated with Z3-3 and *E. coli* DH5α (B), positive control treated with Z3-3 and *B. thuringiensis* subsp. *israelensis* B23 (C), and experimental group treated with Z3-3 and strain XN-10 (D). Macerated tissues of potato slices accounted for 35.3%, 30.7%, 7.0%, and 6.3% in groups A, B, C, and D, respectively. The area of macerated tissues reached up to 31.3%, 30.3%, 12.0%, and 9.0% on carrots in groups A, B, C, and D, respectively. The numbers on Chinese cabbage were 52.0%, 53.0%, 3.3%, and 1.7%, respectively. These data manifested the decay degree of the group inoculated with Z3-3, and strain XN-10 had a lower decay than the group inoculated only with Z3-3. Thus, strain XN-10 had significant inhibitory effects against soft rot on manifold crops caused by Z3-3.

### 3.4. Metabolic Products and Degradation Pathway of AHL

To explore the degradation products and pathways of AHL, samples were collected at different times and analyzed and identified by GC-MS with a library database. The GC peaks of degradation products are shown in Figure S3. By comparing the gas chromatograms of the samples at different times to the control group, three peaks were initially marked as compounds A, B, and C. After background correction, the mass spectra of each chromatographic peak were analyzed and identified on the basis of the similarity of their fragment retention time (RT) and molecular ions to the corresponding authentic compounds in the NIST library database. Compound A was significantly detected in all samples, which eluted at 3.518 min, displayed a characteristic mass fragment [M+] at m/z 143.0 with major fragment ions at *m*/*z* = 57.1 and 99.1 (Figure S4a), and was identified as C6HSL (Figure S5a). Compound B was detected in 6 and 12 h samples, eluted at RT of 3.695 min, showing a prominent protonated molecular ion at *m*/*z* 74.1 (Figure S4b), and was confirmed as *N*-cyclohexyl-propanamide (Figure S5b). However, compound B was not detected in the sample at 24 h, but compound C was found to have a RT of 3.698 min, showing a prominent protonated molecular ion at *m*/*z* 74.1 with major fragment ions at *m*/*z* = 43.0 and 99.1 (Figure S4c) and was characterized as pentanoic acid, 4-methyl, methyl ester (Figure S5c).

**Figure 9:**
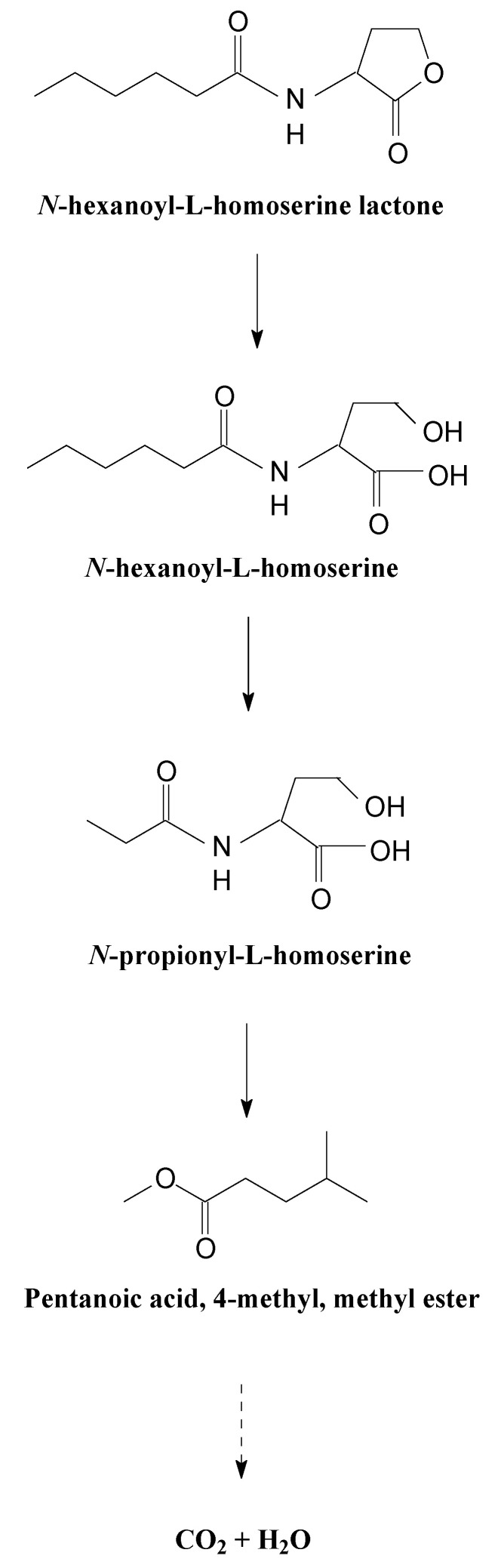
Proposed *N*-acyl-homoserine lactone (AHL) degradation pathway in strain XN-10.

A new metabolic pathway of AHL in strain XN-10 was proposed based on the analysis of AHL chemical structures and metabolites. As shown in Figure 9, AHL was initially degraded by hydrolysis of its ester ring to yield *N*-hexanoyl-L-homoserine. *N*-hexanoyl-L-homoserine can be further degraded by dehydroxylation into two main metabolites: *N*-cyclohexyl-propanamide and pentanoic acid, 4-methyl, methyl ester, respectively [28]. These intermediate metabolites can be further degraded by breaking peptide bonds and ester bonds. Eventually, AHL was degraded and metabolized by strain XN-10 without any persistent accumulative product. This is the first report of an AHL degradation pathway by hydrolysis and dehydroxylation in a microorganism, which we propose to be of vital importance in AHL degradation.

## 4. Discussion

Plant diseases are affected by three interdependent factors, including synchronized activity of regulation networks, virulence factors, and infection processes of bacterial pathogens [36]. Overuse of antibiotics, widely used and recognized as the most effective antimicrobial agents for curing diseases caused by pathogenic bacteria in current production applications, has led to environmental pollution and high occurrence of bacterial resistance [37]. Numerous bacteria serving as biocontrol agents to manage plant diseases have been verified [27,31,35,38]. However, only a few could prevent and control crops from pathogenic bacteria, through interrupting QS systems. To meet this requirement, bacterial isolate XN-10 from agricultural contaminated soil with remarkable AHL degrading activity was identified as *Acinetobacter* sp. It is a Gram-negative, short rod shaped, and non-flagellated bacterium. To the best of our knowledge, there are few reports of AHL degradation from genus *Acinetobacter* [28,39,40,41]. For the first time, this study proved that *Acinetobacter* sp. can reduce the severity of soft rot disease caused by *Pcc* by degrading the QS signals.

QQ is a method for interrupting QS and can be achieved in many different pathways. QQ has recently been recognized as a novel and potential strategy for disease control in aquaculture, agriculture, and human healthcare [42,43,44,45]. Theoretically, there are many ways to achieve QQ; however, certain methods are not feasible. For example, changing environmental conditions (pH and temperature) and reconstructing bacterial metabolic pathways are infeasible in practical application [45]. Application of QS inhibitors is more feasible and applicable compared to other QQ ways against pathogens [45]. QS inhibitors fall into two categories: QQ enzymes and certain chemicals extracted from natural resources or obtained by chemical synthesis [46,47,48,49]. In isolating QQ enzymes from bacteria, it has been confirmed that there are a variety of strains presenting QS signal inactivation capabilities in different microbial genera [50,51,52].

However, QQ strains directly applied to combat pathogenic bacterium have not been fully explored. In this article, we demonstrated that *Acinetobacter* sp. strain XN-10 was able to reduce the severity of soft rot disease caused by *Pcc* in potatoes, Chinese cabbage, and carrots. Furthermore, adverse effects of isolate XN-10 on the host plants were not observed during this study. This study demonstrated that isolate XN-10 is able to serve as a broad-spectrum biocontrol agent and control plant diseases caused by AHL-dependent pathogens.

Nevertheless, several interesting phenomena should be considered. Firstly, QQ strains cannot specifically inactivate QS signals of certain bacteria. Consequently, unnatural phenomena and states of beneficial host-associated microbes may appear and generate a range of reactions [45]. Another notable potential issue is the development of resistance. It is widely accepted that resistance is less likely to develop, because QQ strains impart less selective pressures. However, resistance attributes to QS inhibitors have already been discovered in laboratory and clinical settings [25,52]. Therefore, it is a considerable and notable phenomenon.

## 5. Conclusions

To sum up the findings, the novel QQ candidate *Acinetobacter* sp. XN-10, presenting highly efficient AHL degradation activity and significant biocontrol activity against *Pcc*, was isolated and characterized. Strain XN-10 demonstrated a remarkable capacity to degrade a wide range of AHLs. In addition, application of strain XN-10 could substantially reduce the disease severity of *Pcc* on potatoes, carrots, and Chinese cabbage. As a promising biocontrol agent, strain XN-10 could be further explored to counter other AHL-dependent bacterial pathogens, such as *Dickeya* sp. and *Pseudomonas aeruginosa*. Furthermore, two major metabolic products, *N*-cyclohexyl-propanamide and pentanoic acid, 4-methyl, methyl ester, were identified during the degradation process. This is the first report of a novel pathway for AHL degradation by hydrolysis and dehydroxylation in a microorganism. The findings from this study not only characterize a highly efficient AHL degradation bacterial isolate for biotechnological application, but they also shed light on the QQ mechanism of *Acinetobacter* sp. strain XN-10. However, further studies, such as with AHL degradation-related enzymes and genes and their interaction with environment, are still needed before XN-10 can be used in field-scale biocontrol applications.

## Supplementary Materials

The following are available online at https://www.mdpi.com/2076-2607/8/8/1100/s1, Figure S1: Morphological characteristics of strain XN-10. (a) Colony morphology of strain XN-10. (b) Scanning electron micrograph of strain XN-10. Figure S2. The residual amount of OHHL at different times detected by HPLC. Panel A is MSM with OHHL alone as a control group; Panels B, C, D, E, and F are the remaining OHHL after utilization by strain XN-10 at 0, 1, 2, 3, and 4 days, respectively. Figure S3: Full scan mass spectrum of the AHL degradation products by strain XN-10. a: 12 h; b: 24 h. A: Compound A; B: Compound B; C: Compound C. Figure S4: Mass spectra of AHL degradation products by strain XN-10. (a) AHL; (b) *N*-Cyclohexyl-propanamide; and (c) Pentanoic acid, 4-methyl, methyl ester. Figure S5: Mass spectra of degradation products of AHL by strain XN-10 in the NIST library database. A: AHL; B: *N*-Cyclohexyl-propanamide; and C: Pentanoic acid, 4-methyl, methyl ester.

## Abbreviations

QS	quorum sensing
QQ	quorum quenching
AHLs	*N*-acyl-homoserine lactones
OHHL	*N*-(3-oxohexanoyl)-L-homoserine lactone
C6HSL	*N*-hexanoyl-L-homoserine lactone
3OC12HSL	*N*-(3-oxododecanoyl)-L-homoserine lactone
3OC8HSL	*N*-(3-oxooctanoyl)-L-homoserine lactone
HPLC	high-performance liquid chromatography
GC-MS	gas chromatography–mass spectrometry
3-OH-PAME	3-hydroxypalmitic acid methyl ester
PQS	2-heptyl-3-hydroxy-4(1*H*)-quinolone
AI-2	autoinducer-2
DSF	diffusible signal factor
IQS	2-(2-hydroxyphenyl)-thiazole-4-carbaldehyde
Pel	pectate lyase
Pnl	pectin lyase
Peh	polygalacturonase
Cel	cellulase
Prt	protease
Amp	antibiotics ampicillin
Carb	carbenicillin
Str	streptomycin
TC	tetracycline
Gen	gentamicin
Cm	chloramphenicol
Kan	kanamycin
Neo	neomycin
