# Supplementary material for: Quorum Quenching in a Novel Acinetobacter sp. XN-10 Bacterial Strain against Pectobacterium carotovorum subsp. carotovorum
Source: Microorganisms. 2020 Jul 23;8(8):1100. doi: 10.3390/microorganisms8081100 (PMC7466008; doi:10.3390/microorganisms8081100)
Supplement: Supplementary file 1 [file microorganisms-08-01100-s001.pdf]

Supplementary materials

Quorum quenching in a novel *Acinetobacter* sp. XN-10 bacterial strain against *Pectobacterium carotovorum* subsp. *carotovorum*

Wenping Zhang[1,2#], Qingqing Luo[1,2#], Yiyin Zhang[1,2#], Xinghui Fan[1,2], Tian Ye[1,2], Sandhya Mishra[1,2], Pankaj Bhatt[1,2], Lianhui Zhang[1,2] , Shaohua Chen[1,2*]

[1]State Key Laboratory for Conservation and Utilization of Subtropical Agro-bioresources, Guangdong Province Key Laboratory of Microbial Signals and Disease Control, Integrative Microbiology Research Centre, South China Agricultural University, Guangzhou 510642, China;
[2]Guangdong Laboratory for Lingnan Modern Agriculture, Guangzhou 510642, China
# These authors contributed equally to this work.
*Correspondence: shchen@scau.edu.cn

a

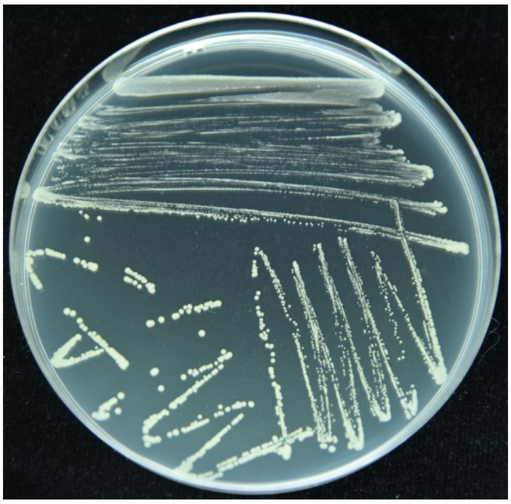

b

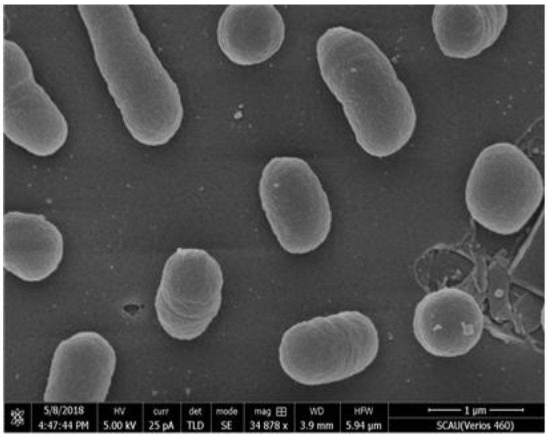

Figure S1. Morphological characteristics of strain XN-10. (a) Colony morphology of strain XN-10. (b) Scanning electron micrograph of strain XN-10.

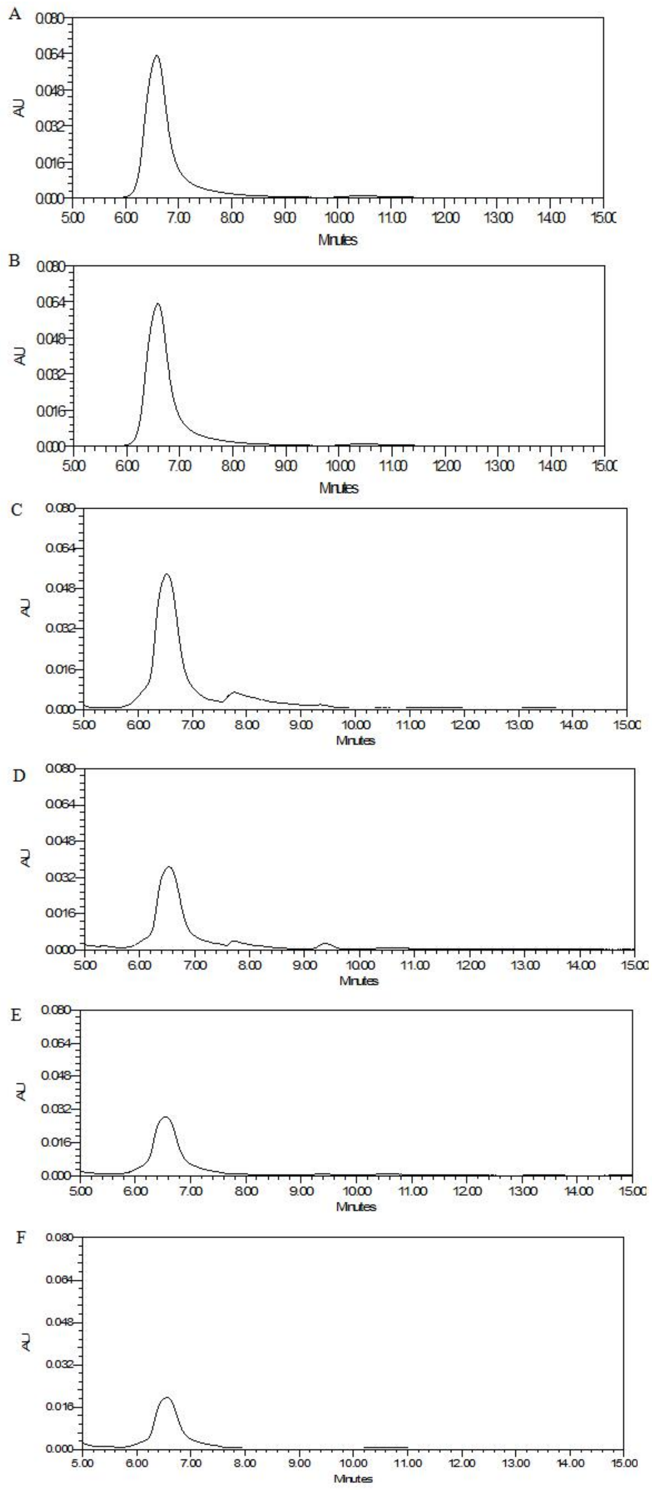

Figure S2. The residual amount of OHHL at different times detected by HPLC. Panel A is MSM with OHHL alone as a control group; Panels B, C, D, E, and F are the remaining OHHL after utilization by strain XN-10 at 0, 1, 2, 3, and 4 days, respectively.

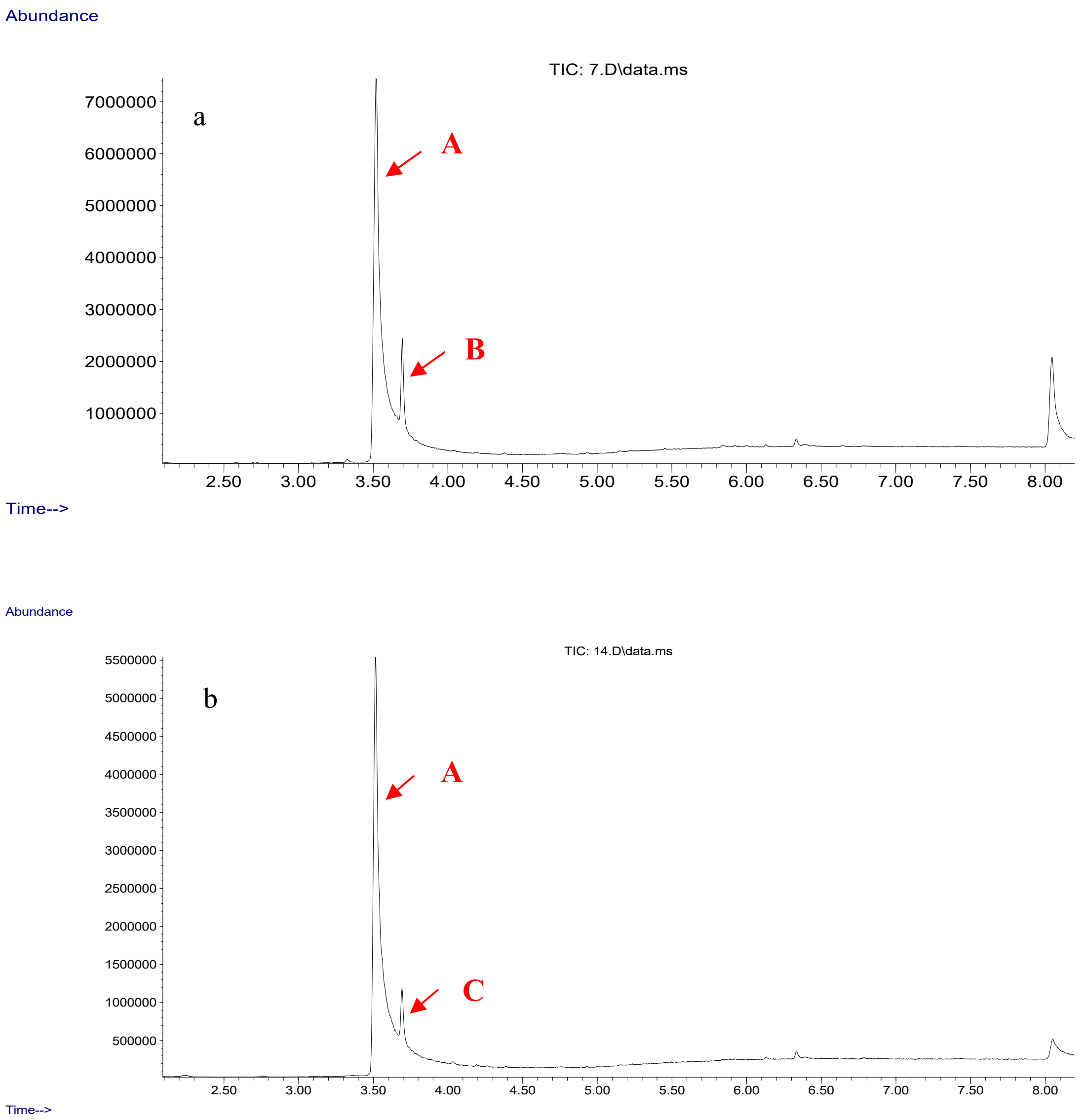

Figure S3. Full scan mass spectrum of the AHL degradation products by strain XN-10. a: 12 h; b: 24 h. A: Compound A; B: Compound B; C: Compound C.

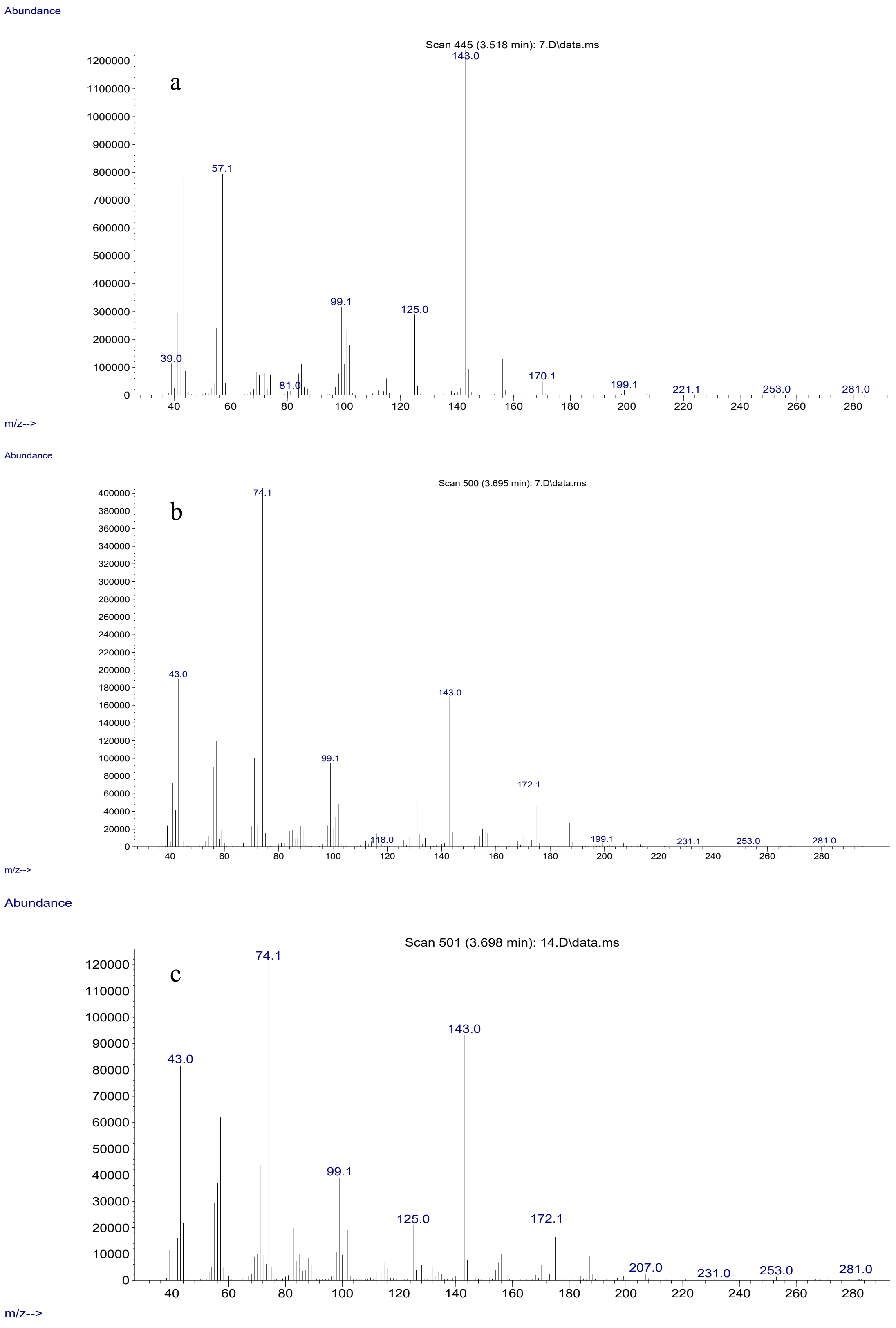

Figure S4. Mass spectra of AHL degradation products by strain XN-10. (a) AHL; (b) *N*-cyclohexyl-propanamide; and (c) Pentanoic acid, 4-methyl, methyl ester.

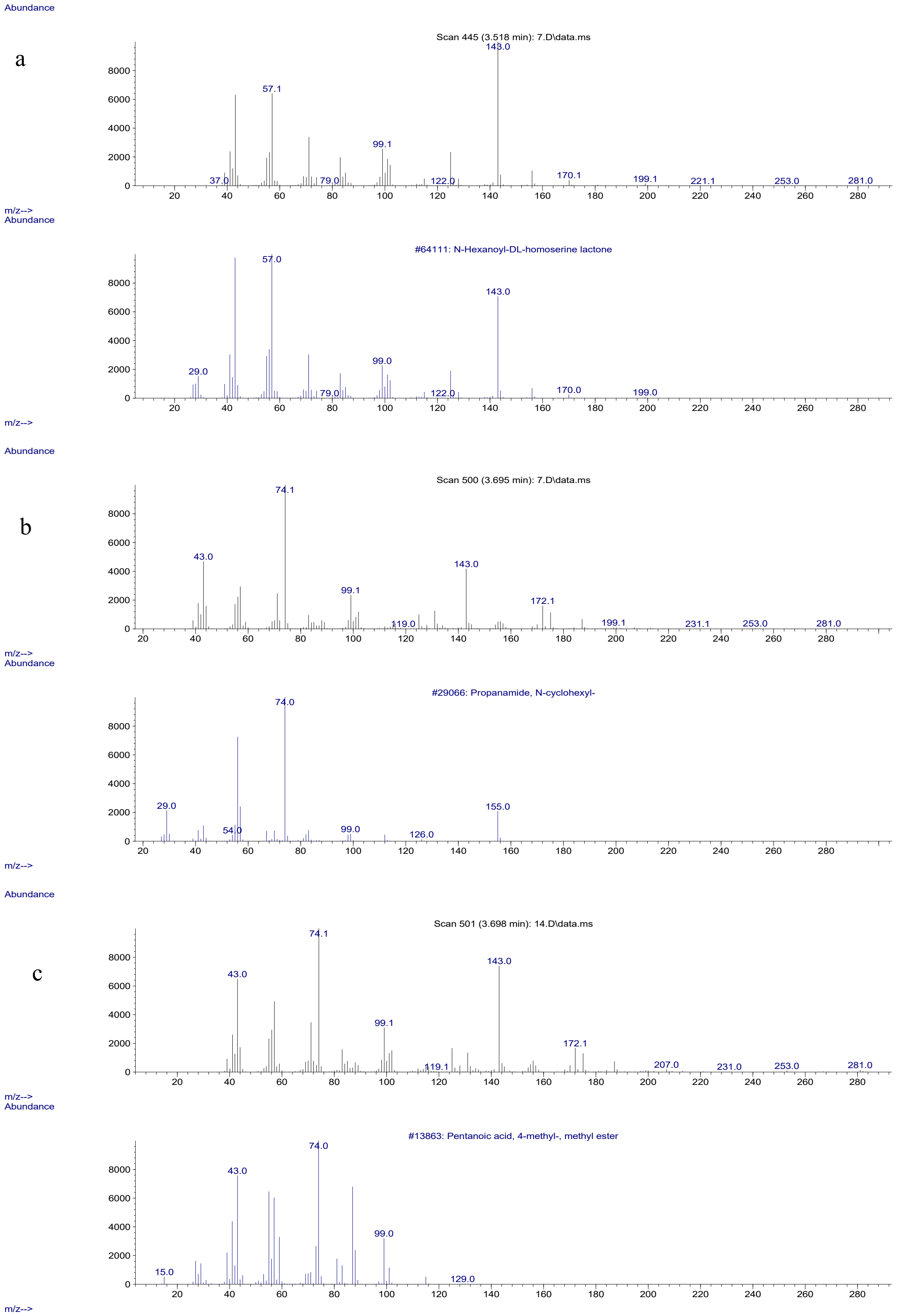

Figure S5. Mass spectra of degradation products of AHL by strain XN-10 in the NIST library database. A: AHL; B: *N*-cyclohexyl-propanamide; and C: Pentanoic acid, 4-methyl, methyl ester.
